# Observations on the Final Cause of Menstruation

**Published:** 1830

**Authors:** James C. Finley


Art.—III. *Observations on the final cause of Menstruation.* By James C. Finley, M. D.

Every thing connected with the subject of generation, has been, at all times, a subject of lively and peculiar interest.—The strong passions which nature, for the accomplishment of her purposes, has implanted in the different sexes, and the tender relations, and the amiable and generous sentiments which arise from the relation in which they stand to each other, have rendered this a subject of inquiry and of curiosity, to every class of society. The student of Natural History would naturally direct his attention to this subject; and some of the most distinguished friends of philosophy, have attempted to illustrate it by their investigations. The inquiry has been pursued with the more avidity, from a delusive hope, that nature has here exposed her mode of workmanship more immediately to our view, and that the veil which she has elsewhere so carefully thrown over her operations, might here perhaps be drawn aside. But she holds out her inducements to investigation, only to elude its efforts, and to lead the bold inquirer aside from the high road of science, into the devious paths of vain speculation. Upon no subject has more labor been bestowed, and upon no subject have the inquiries of the most powerful minds terminated in results so perfectly unsatisfactory. Vain hypotheses, and unfounded speculations, have followed each other in rapid succession, and for a time, served to gratify the inquisitive mind of man with the specious of knowledge, until some new theorist has given currency to a rival system, rather by pulling down the frail fabric of his predecessor, than by establishing one more substantial of his own.

It is upon the attention of the physiologist, and the physician, that this subject has the strongest claims. Upon the former, on account of the powerful influence which the organs of generation exert over the whole animal economy: an influence, which, commencing at a very early period of life, and at a time when they are so little developed, as scarcely to convey any idea of the important office they are afterward to subserve, is secretly modifying the character, form, and disposition of the sex, and imparting to each those powers, sentiments, and feelings requisite to qualify them for the part they are afterward to perform. These organs exert, both on the male and on the female, a very powerful influence, but of a very opposite kind. To the frame of the former it gives the structure, and imparts the vigor and strength, necessary for the part he is to act in the bustling and active scenes of life. They give to the latter that structure which is best adapted to parturition, and develope those organs which must supply nourishment to her offspring.

Their influence on the moral, active, and intellectual powers is equally striking. In both sexes they tend to refine and improve these faculties. To the one they give courage and enterprise, combined with tenderness and generosity; to the other those interesting manners and amiable virtues, which are requisite to win the affections and soften the cares of her husband, and to provide for the comfort and to cultivate the moral and intellectual faculties of her children.

To the attention of Physicians, this subject is recommended, by the fact that the important office these organs are destined to subserve in the animal economy, their great sensibility, and the numerous sympathies by which they are connected with the rest of the system, give them a predominating influence, especially in the female sex, through a great period of life, and prove so frequent an avenue of disease, as to call for the exercise of professional skill, under circumstances calculated to elicit the most generous feelings of our nature.

A few physiologists, of late years, warned by the failure of their predecessors, have pursued their researches in the true spirit of modern philosophy, and have, perhaps, acquired all the information that simple observation can convey. Much, however, remains to be done in the way of analogical illustration, and we have no doubt that in future, much light will, in this way, be thrown upon the obscure physiology of these organs.

Under the influence of a feeling of this kind, we are disposed to offer some remarks upon the uses of menstruation. The ill success of our predecessors is certainly a very powerful reason why we should tread in their steps with caution, and confine ourselves to the surer regions of experiment and observation, rather than enter into a field where we can be guided only by the uncertain light of analogy; but the efforts of many of the most illustrious ornaments of science will sanction our efforts, and, if it should be necessary, mitigate the mortification of a failure. The speculations in which we are engaged are not indeed calculated very directly to advance the interests of the healing art; but truth is always desirable, and correct views, at once prevent the mind from wandering in pursuit of error, and prepare it for those exertions which are more immediately connected with the exercise of professional duty.

This periodical discharge is peculiar to the human female. Of the numerous and unsatisfactory hypotheses which have been formed in explanation of its final cause, we shall notice but three.

Galen supposed that after the completion of the growth of the body, the fluids no longer required for the completion of the organs, existed in excess, and that nature was supposed to keep up this supply in the female sex, as a provision for the nourishment of the foetus. In order, however, to avoid any injurious consequences from plethora, an outlet was to be provided until gestation should appropriate the surplus fluids to their proper objects. This outlet is generally found in the vessels of the uterus, yet when nature is prevented by accidental circumstances, from relieving herself in this manner, the vessels of other surfaces may assume a vicarious office, and the system be relieved by a discharge from the breasts, &c. &c. Numerous facts, so well authenticated as to leave no room for doubt, do indeed establish the fact, that discharges of this nature, observing the regular menstrual period, do take place, but they are comparatively so rare, that they are to be looked upon rather as anomalies, which the present state of physiological science does not permit us to explain, than as affording any substantial support to this opinion. But we will defer our objections to this opinion, until we come to the consideration of the views of Dr. Cullen.

This author advanced an idea very analogous to that of Galen, but founded on the supposition of local, instead of general plethora. As the growth of each organ in the body is successively completed, he supposes that the excess of fluids thus left in the system, is diverted to other organs. The uterine system, coming last to perfection, is left in a plethoric condition, which is relieved by an effusion from the mouths of the vessels opening upon the surface of the uterus, by an effort of nature to which we have many analogies in the history of the diseases of the animal economy. The discharge thus produced to relieve a particular contingency, is afterward kept up by that tendency to periodical returns, which we meet with in many of the animal organs, and especially in the uterus.

The errors of both these opinions, we think, may be shown by a very similar train of reasoning.

1. Menstruation is not a diseased, but a healthful action of the female system, designed to answer some important indication in generation, and so essential to this process, that impregnation may be said never to take place when this is deranged ; and hence it is guarded by nature with such peculiar care, that the slightest derangement and irregularity in this respect, throws the whole system into confusion. It sympathizes also with the system in the deranged functions of the other organs, and is very frequently suppressed when the other secretions are suspended; although a very plethoric condition of the system may prevail ; and, on the other hand, when the system is worn down by some wasting disease, it frequently continues, provided the general integrity of the functions of the organs be preserved, until the powers of life are nearly exhausted.

2. It cannot depend upon plethora, because at the very period it makes it appearance, and for a considerable time afterward, the whole system is growing with great rapidity, and in consequence of the new appetites, passions, and emotions, which are at this time called into action, is subjected to a wear and tear unknown at any other period of life.

3. Lastly, the whole history of the animal economy shews, that in its healthful condition, there is a strict connexion between its wants and the supply of nutriment. If an additional supply becomes necessary in consequence of any accidental drain, or for the growth of an organ, the nutritive vessels are temporarily called into increased action, to meet the demand ; and if the change produced in the system be not so great as to induce a state of disease, when the necessity for this increased action ceases gradually accommodate themselves to the change of circumstances. If, therefore, symptoms of plethora occasionall appear at the period of puberty, it is not that they attend as a necessary consequence, upon the completion of the animal growth ; but that the nutritive vessels, called into unusual activity to meet the present wants of the system, occasionally overact their part ; or that the increase of sensibility and vascular action, which invariably attend the growth of the body, render it, at this time, peculiarly liable to disease.

The opinion of Mr. Charles Bell is, we think, entitled to more credit. He supposes that a periodical excitement takes place in the system, for the purpose of maturing the ovum ; and that the flow of blood is intended to allay the excited state of the uterine vessels. This opinion, which is strengthened by the previous symptoms, and by dissections, derives additional confirmation from other considerations.

In the lower orders of animals, the union of the sexes succeeds the excitement of the uterine system almost invariably, and the regular course of events indicated by nature, follows each other in uninterrupted succession. If this should not be the case, the short periodical excitement of the organs, followed by long intervals of perfect quiescence, affords but a slight opportunity for structural derangement to take place. In the human family, the uterine system is kept in a state of almost continual excitement, while the moral considerations, which result from the social condition in which it was evidently the design of the Creator that man should be placed, prevent that train of events by which the evil consequences of this excitement might be obviated. Hence, in females that are barren, and more especially, in those that live in a state celibacy, a foundation is laid for various structural diseases, cancer, &c. &c. Nature, both in the natural and moral world, frequently employs agents to modify the excessive action of her instruments, and the animal propensities arising from the healthy action of the generative organs, proceeds so nearly to the extent of disease, that an agent of this kind may be considered as far from being unnecessary. Above all, there is a very close analogy between this discharge and the remedies to which, on similar occasions, nature usually resorts.

To this hypothesis, for, in the absence of any direct and conclusive evidence, it can be regarded only in the light of an hypothesis, we have no satisfactory objection to offer; and as its admission will not militate against the view we have taken, we shall avoid its further discussion, simply remarking, that we consider the suggestion as entitled to more consideration than any that has hitherto been offered.

This discharge, as we have already remarked, occurs with too much regularity in almost every condition of health, is too intimately connected with the fruitful condition of these organs, and is preserved by nature with too much care, to be looked upon either as the result of disease, or as destined to perform a secondary part in preparing the uterine system for the performance of its functions.

We shall, therefore, proceed to inquire into the peculiarities which characterize the process of generation in women, with the hope of finding some use for this discharge, more in accordance with the importance which nature has attached to it.

Upon inquiring into the manner in which the fœtus in viviparous animals is connected with the uterus of the mother, we find, that among many individual peculiarities, three general modes of union may be observed.

1. In the first, the membranes enveloping the ovum, have an immediate vascular connexion with the uterus, without the intervention of a placenta.

2. In the second, the ovum is connected to the uterus by means of one, or more placentæ, the fœtal and maternal parts of which are distinct.

3. The third variety is met with in man and the monkey tribes, in which is formed a *deciduous membrane secreted by the vessels of the internal surface of the uterus* that, with the whole placenta, is thrown off at parturition.

In this peculiarity we are disposed to seek for the explanation we have in view. All the mucous membranes, under a certain grade of irritation, appear to have the power of secreting a membrane of coagulable lymph imperfectly organized. In the present instance, the functions of the organ require that a membrane should be formed with a more perfect organization, and under a degree of irritation that should not exceed the healthy grade. For this purpose, a set of vessels would appear to be necessary, that would open readily upon the surface of the uterus, and secrete a vascular and organized membrane, under that slight degree of irritation which the local determination consequent upon conception would produce.

It is peculiar to the females of our species, that they continue constantly in a condition favorable for conception, during the whole period from purberty to the time that the power of reproduction entirely fails. In order that these vessels may not suffer (as other parts of the vascular system do when their action is not immediately called for in the system) from the inactivity in which they are often kept, in consequence of moral obligations and other circumstances of an accidental nature, it would appear to be necessary that they should occasionally be called into exercise. This discharge we suppose to be the means to which nature has recourse for this purpose.

In confirmation of this opinion, we find that the uterus, in a state of disease, frequently forms this membrane under a state of irritation so slight, as to produce no general febrile action in the system. Dr. Eberle, indeed, supposes that this adventitious membrane is always the result of irritation. Granting this to be the case, the irritation, since it is frequently entirely local in its character, must be very slight; and if a greater degree of febrile action occasionally attends, it may rather be attributed to the the disturbance produced by the derangement of the functions of so important an organ as the uterus, than to the inflammatory character of the local disease.

If it be inquired why this discharge is peculiar to the human family, and is not met with in other animals that secrete the deciduous membrane, we reply :

1. All other animals have periodical seasons when their organs are prepared for conception. Conception and gestation almost invariably follow this state of preparation. If accidental circumstances should prevent this, they relapse again into their former state of inactivity, until the periodical excitement again returns. Thus, even when their natural habits are subverted by domestication, their generative system through a great portion of the year, is as completely dormant, as before puberty ; and in many animals,requires as remarkable a development to prepare it for conception, as takes place at that period. May we not, therefore, reasonably suppose, that the different condition which Providence has imposed upon woman, would require a corresponding modification in the structure and physiology of these organs.

2. To those who are dissatisfied with this explanation, we would remark, that the structure and organization of animal life is more complicated, in proportion as we advance in the scale of animated nature. Nature has given to most animals sufficient powers of self preservation, and reproduction, to enable them to exist comfortably in the sphere in which she has designed them to move, and to continue their species. Beyond this, she has been sparing of her gifts. Accordingly, we find that those which possess organs calculated to enable them to preserve their existence in one element, are either destitute of those organs which would qualify them to move in another, or possess them in a very imperfect condition. Those animals whose imbecility exposes them to the attacks of the more rapacious species, bring forth a numerous progeny after a short and easy gestation. In those that are protected by their size, strength, or courage, the perfection and birth of the fœtus is accomplished by a slower and more laborious process. The intelligence of man gives him a very great superiority over other animals, and hence in conformity with this universal law, his organs, except where a more perfect organization is necessary to develop, or subserve his intellectual character, are comparatively imperfect. This imperfection is peculiarly to be witnessed in those organs, which are concerned in the reproduction of the species. The very structure and position of the uterus exposes the female to many dangers and inconveniences in gestation, from which the inferior animals are free; parturition is more painful and hazardous, and as an extension of the same principle, the infant is more helpless, continues longer in a state of dependence, and requires more education to enable it to provide for its own wants, than we meet with in other animals. Why, therefore, should we be surprised if the same imperfection should attend this part of the system, in its unimpregnated state, and if in preparing these organs for conception, and in preserving them in that condition, the female should be subjected to inconveniences from which other animals are exempt.

We have taken for granted, throughout this essay, upon the authority of Mr. Lawrence, that this evacuation is not met with in the monkey tribes. Mr. L. states, that the popular belief, which has been countenanced by some distinguished physicians, has risen from the fact of the peculiar liability of these animals to uterine hemorrhage. Either opinion, however, is favorable to our view, inasmuch as it tends to show that a provision has been made for the secretion of the decidua, by a set of vessels, larger, and opening upon the surface of the uterus more readily, than occurs in other animals.

